# Analysis of risk factors and clinical implications for diabetes in first-degree relatives in the northeastern region of China

**DOI:** 10.3389/fendo.2024.1385583

**Published:** 2024-06-11

**Authors:** Zhenglin He, Hayato Yamana, Hideo Yasunaga, Hongjun Li, Xue Wang

**Affiliations:** ^1^ China-Japan Union Hospital of Jilin University, Jilin University, Changchun, China; ^2^ Data Science Center, Jichi Medical University, Shimotsuke, Japan; ^3^ Department of Clinical Data Management and Research, Clinical Research Center, National Hospital Organization Headquarters, Meguro, Japan; ^4^ Department of Clinical Epidemiology and Health Economics, School of Public Health, The University of Tokyo, Bunkyo, Japan; ^5^ Health Management Medical Center, China-Japan Union Hospital of Jilin University, Changchun, China; ^6^ Department of Clinical Nutrition, China-Japan Union Hospital of Jilin University, Changchun, China

**Keywords:** diabetes millitus, family history, diagnosed T2DM, undiagnosed T2DM, uncontrolled T2DM

## Abstract

**Background:**

The prevalence of diabetes has risen fast with a considerable weighted prevalence of undiagnosed diabetes or uncontrolled diabetes. Then it becomes more necessary to timely screen out and monitor high-risk populations who are likely to be ignored during the COVID-19 pandemic. To classify and find the common risks of undiagnosed diabetes and uncontrolled diabetes, it’s beneficial to put specific risk control measures into effect for comprehensive primary care. Especially, there is a need for accurate yet accessible prediction models.

**Objective:**

Based on a cross-sectional study and secondary analysis on the health examination held in Changchun City (2016), we aimed to evaluate the factors associated with hyperglycemia, analyze the management status of T2DM, and determine the best cutoff value of incidence of diabetes in the first-degree relatives to suggest the necessity of early diagnosis of diabetes after first screening.

**Results:**

A total of 5658 volunteers were analyzed. Prevalence of T2DM and impaired fasting glucose were 8.4% (n=477) and 11.5% (n=648), respectively. There were 925 participants (16.3%) with a family history of T2DM in their first-degree relatives. Multivariable analysis demonstrated that family history was associated with hyperglycemia. Among the 477 patients with T2DM, 40.9% had not been previously diagnosed. The predictive equation was calculated with the following logistic regression parameters with 0.71 (95% CI: 0.67–0.76) of the area under the ROC curve, 64.0% of sensitivity and 29% of specificity (*P* < 0.001): *P* = \frac{1}{1 + e^{-z}}, where z = -3.08 + [0.89 (Family history-group) + 0.69 (age-group)+ 0.25 (BMI-group)]. Positive family history was associated with the diagnosis of T2DM, but not glucose level in the diagnosed patients. The best cutoff value of incidence of diabetes in the first-degree relatives was 9.55% (*P* < 0.001).

**Conclusions:**

Family history of diabetes was independently associated with glucose dysfunction. Classification by the first-degree relatives with diabetes is prominent for targeting high-risk population. Meanwhile, positive family history of diabetes was associated with diabetes being diagnosed rather than the glycemic control in patients who had been diagnosed. It’s necessary to emphasize the linkage between early diagnosis and positive family history for high proportions of undiagnosed T2DM.

## 1 Introduction

Over the past decades, prevalence of diabetes has risen fast in low- and middle-income countries, but its developing trends are still not very pessimistic with a weighted prevalence of total diabetes (12.8%), self-reported diabetes (6.0%), newly diagnosed diabetes (6.8%), and prediabetes (35.2%) (1). It is worth considering that more than 80% of cases of undiagnosed diabetes are in low- and middle-income countries (2, 3). Since 2020, however, the global medical resource allocation has been greatly affected by the novel coronavirus disease (COVID-19) pandemic (4, 5). Then it becomes more necessary to timely screen out and monitor high-risk populations likely to be ignored during the COVID-19 pandemic. To classify and find the common risks of undiagnosed diabetes and uncontrolled diabetes, it is beneficial to put the specific risk control measures into effect directly for comprehensive primary care.

As the guidelines that the World Health Organization (WHO) stated, it is crucial to consider the condition of prediabetes and uncontrolled diabetes (6). Studies from Canada and Bangladesh reported that more than half of the people with diabetes remained undiagnosed (7). Nationwide in China, the proportion of patients with undiagnosed T2DM reached 75% in 2003 and 32.5% in 2010 (8, 9). The adjusted prevalence of metabolic syndrome was reported to be higher in the northern China (15.0%) than that of the southern China (6.8%). Among adults in China, the estimated overall prevalence of diabetes was 10.9%, and that for prediabetes has reached 35.7% (10). Moreover, diabetes and impaired fasting glucose were highly prevalent among adults in Northeast China (11). Changchun, the urbanizing Northeast Chinese city with a population of about 9.07 million, its prevalence of diagnosed and undiagnosed type 2 diabetes mellitus (T2DM), factors related to hyperglycemia, and management status of T2DM have not been clarified yet.

Identification of previously undiagnosed and uncontrolled T2DM patients is as consequential as continually recognizing genetic risk factors and modifiable unhealthy lifestyles for diabetes prevention and control (12). It depends on the popularization of health education and the acceptance of self-responsibility among the people who have been informed of T2DM or related risk factors, otherwise the control goals assigned with the target populations will be meaningless. It has been assumed that rapid screening and early treatment should be enhanced in the population with positive family history of T2DM (13–16). Given the difference in cultural, health behavioral, socioeconomic, and demographic among diabetes patients with different family histories, diagnosis and treatment would naturally vary widely.

Based on a cross-sectional study and secondary analysis on the health examination held in Changchun City (2016), we evaluated the factors associated with hyperglycemia and analyzed the management status of T2DM, aiming to determine the best cutoff value of incidence of diabetes in the first-degree relatives to suggest necessary early diagnosis of diabetes after first screening. We assumed that positive family history is an independent risk factor for hyperglycemia. Also, we hypothesized that people with positive family history would be more likely to have been diagnosed with T2DM and have controlled the disease more effectively.

## 2 Methods

### 2.1 Participants and study design

The study was approved by the China-Japan Union Hospital of Jilin University Ethics Committee for Human Subjects in Research (reference number 16-ks-007). Between every Saturday and Sunday in September 2016 and March 2017, volunteers aged ≥18 years who had lived in the seven urban districts for at least six months were recruited to take routine medical check-ups. All participants provided written informed consent before taking examinations and investigations. We preliminarily considered the sampling opportunity and quantity to ensure sufficient and representative number of participants. Based on a systematic review from 2000 to 2010, the overall adjusted prevalence of diabetes was estimated to have increased to 9.95% at the provincial level (17). To show a prevalence of 9.95% with a permissible error of 10% and a significance level of 0.05, the required sample size was estimated to be 3600. Allowing for a 20% withdraw rate, the sample size was enlarged to 4320. In total, 5658 participants were included in this study.

The following variables were analyzed: demographic characteristics from questionnaires at one-to-one interviews (age, monthly income, educational level, physical activity status, drinking history, smoking history, family members and diabetic histories); physical indices (body weight, height, and waist circumference); health examination including electrocardiography (ECG), abdominal ultrasonography examination, and biochemical indices including total cholesterol (TC), triglyceride (TG), high-density lipoprotein (HDL), low-density lipoprotein (LDL) and FPG.

### 2.2 Definition

T2DM was diagnosed based on the criteria of the American Diabetes Association (ADA): a FPG ≥7.0 mmol/L and/or a self-reported history of having been diagnosed by physicians. IFG was defined by 5.6 mmol/L < FPG < 7.0 mmol/L (ADA, 2003) without a self-reported T2DM. IFG and T2DM were considered as hyperglycemia in this study. Fatty liver and ECG information was confirmed by clinicians. Blood sample analysis was conducted with standard laboratory methods in the clinical laboratory center of China-Japan Union Hospital. Body Mass Index (BMI) was categorized into four groups according to the WHO Asia criteria of underweight, normal weight, overweight, and obesity (<18.5, 18.5–23.0, 23.0–25.0, and ≥25.0 kg/m^2^, respectively). Three lipid indices were calculated: TG/HDL, LDL/HDL, and TC/HDL (18, 19).

Smoking status, drinking status, and physical exercise were assessed by the interview by trained nurses. Smoking status was categorized as follows: non-smoker, passive smoker, ex-smoker, and smoker. Drinking status was categorized as follows: non-drinking, standard drinking (12 g/day of alcohol), moderate drinking (less than two drinks a day for a man and no more than one for woman), and hazardous drinking (any greater amount). Physical exercise was categorized into three groups: regular (taking physical exercise at least 30 minutes at moderate/vigorous intensity and no less than three times per week), occasional (one to three times per week), and less/never (less than once per week).

Exposure to family history of T2DM was categorized as ‘+’ when the respondents were aware of one of their first-degree relatives, including parents, brothers and sisters, who had or died of diabetes and ‘++’ when there were more than one such first-degree relatives. All others were defined as unexposed (‘-’). Meanwhile, all people with diabetes in the first relatives were recorded.

### 2.3 Statistical analysis

All statistical analyses were conducted with SPSS software (21.0, IBM Co., Armonk, NY, USA). For summary statistics, proportions were used for categorical variables. Means with standard deviations and medians with inter-quartile ranges were used for continuous variables. We then conducted two analyses. Firstly, characteristics of participants with hyperglycemia (IFG or T2DM) were compared with those of normo-glycemic participants. Secondly, among T2DM participants, the previously diagnosed patients were compared with the previously undiagnosed participants who were newly diagnosed in this study.

The categorical variables were evaluated by Pearson’s chi-squared test and the chi-squared test for trend. Mann-Whitney U test was used for analyzing continuous variables of non-normal distributions determined by one-sample Kolmogorov-Smirnov test. The variables which were significant (*P <*0.10) at chi-square test or Mann-Whitney U test were entered as potential independent variables in the multivariable logistic regression analyses. The optimal cutoff value of incidence of diabetes in the first-degree relatives was determined using the Receiver Operator Characteristic (ROC) curve. A two-sided p-value < 0.05 was considered statistically significant.

## 3 Results

### 3.1 General characteristics

Among the 5870 study participants, 172 participants had missing values on items (family history, smoking and drinking status, etc.) and 40 did not agree to receive blood tests or abdominal ultrasonography. Ultimately, 5658 participants (96.8%) were included for further analysis.

**Figure 1:**
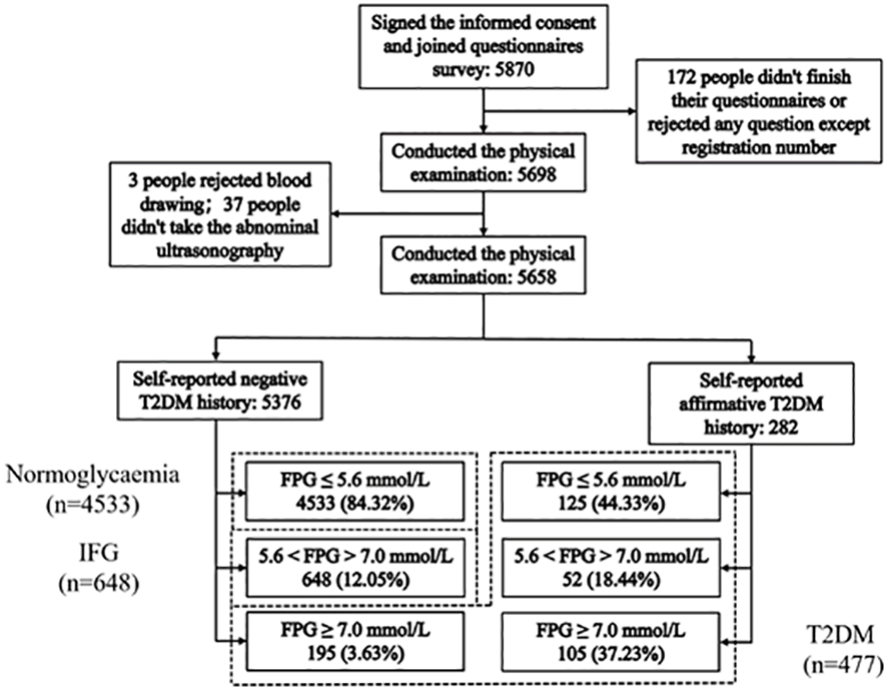
Flowchart showing the participant selection and the final participants. 477 T2DM: FPG ≥7.0 mmol/L (195) and self-reported history of having been diagnosed by physicians (282); 648 IFG: 5.6 mmol/L < FPG < 7.0 mmol/L (ADA, 2003) without a self-reported T2DM; 4533 Normoglycaemia: FPG ≤ 5.6 mmol/L without a self-reported T2DM.

The self-reported personal medical history showed that there were 282 participants previously diagnosed with T2DM (5.0%), including 105 patients (37.2%) with a high glucose level (≥7.0 mmol/L). Simultaneously, the 5376 participants who denied the diagnosis with T2DM (95.0%) included 648 participants (11.5%) with IFG (5.6–7.0mmol/L) and 195 participants (40.9%) with diabetes (≥7.0 mmol/L). Therefore, the overall prevalence of hyperglycemia was 19.9% (1125/5658) with a T2DM prevalence of 8.4% and IFG prevalence of 11.5%, as shown in **Figure 1**. Of the 5658 participants, 925 participants (16.3%) had one or more first-degree relatives with T2DM. The prevalence of hyperglycemia among these participants was 24.9% (230/925).

### 3.2 Factors associated with IFG and T2DM

As manifested in **Table 1**, the following variables showed significant associations with hyperglycemia in bivariate analyses (*P <*0.001): age, sex, family history, income level, educational level, smoking status, drinking status, physical activity, preference for sweetened food, fatty liver, ECG, BMI, waist circumference, TC, TG, TC/HDL, and TG/HDL. The level of HDL (p=0.06), LDL (p=0.09), and LDL/HDL (*P* =0.95) were not associated with hyperglycemia.

**Table 1-1:** Participant characteristics and glycemic status.

Characteristic	Total (N=5658) n (%)	Normo-glycaemia (N=4533) n (%)	IFG + DM (N=1125) n (%)	*P*
Family history of diabetes				
>1 family member (++)	91 (1.6)	47 (1.0)	44 (3.9)	<0.001
1 family member (+)	834 (14.7)	648 (14.3)	186 (16.5)	
None (-)	4733 (83.7)	3838 (84.7)	895 (79.6)	
Sex				
Male	3488 (61.6)	2604 (57.4)	884 (78.6)	<0.001
Female	2176 (38.4)	1929 (42.6)	241 (21.4)	
Educational level				
≥College degree	1411 (24.9)	1165 (25.7)	246 (21.9)	<0.001
High school or equivalent	3528 (62.4)	2853 (62.9)	675 (60.0)	
< High school	719 (12.7)	515 (11.4)	204 (18.1)	
Income level				
≥¥3,000	838 (14.8)	612 (14.8)	226 (20.1)	<0.001
¥2,000-¥3,000	3864 (68.3)	3114 (68.3)	750 (66.7)	
<¥2,000	956 (16.9)	807 (16.9)	149 (13.2)	
Preference for sweetened food				
Yes	3336 (59.0)	2576 (56.8)	760 (67.6)	<0.001
No	2322 (410)	1957 (43.2)	365 (32.4)	
Electrocardiogram				
Abnormal	749 (13.5)	551 (12.2)	198 (17.6)	<0.001
Normal	4909 (86.5)	3982 (87.8)	927 (82.4)	
Fatty liver				
Yes	2199 (38.9)	1635 (36.1)	564 (50.1)	<0.001
No	3459 (61.1)	2899 (63.9)	561 (46.9)	
Age (years)				
≥ 60	112 (2.0)	72 (1.6)	40 (3.6)	<0.001
50∼59	1141 (20.2)	756 (16.7)	385 (34.2)	
40∼49	2190 (38.7)	1851 (40.8)	339 (30.1)	
<40	2215 (39.1)	1854 (40.9)	361 (32.1)	
Smoking status				
Smoker	2143 (37.9)	1790 (39.5)	353 (31.4)	<0.001
Passive smoker	1293 (22.9)	1074 (23.7)	219 (19.5)	
Ex-smoker	283 (5.0)	199 (4.4)	84 (7.5)	
Non-smoker	1939 (34.3)	1470 (32.4)	469 (41.7)	
Drinking status				
Hazardous	415 (7.3)	302 (6.7)	113 (10.0)	<0.001
Moderate	1065 (18.8)	810 (17.9)	255 (22.7)	
Standard	973 (17.2)	763 (16.8)	210 (18.7)	
Non-drinking	3205 (56.7)	2658 (58.6)	547 (48.6)	
Physical exercise				
Occasional	1292 (22.0)	1060 (22.0)	112 (21.2)	<0.001
Regular	3133 (53.4)	2663 (55.2)	236 (44.6)	
Less/never	1445 (24.6)	1097 (22.8)	181 (34.2)	
Body mass index (kg/m^2^)				
≥25	2126 (37.6)	1589 (35.1)	537 (47.7)	<0.001
23.0 ∼ 24.9	1261 (22.3)	1003 (22.1)	258 (22.9)	
18.5 ∼ 22.9	2081 (36.8)	1770 (39.0)	311 (27.6)	
<18.5	190 (3.4)	171 (3.8)	19 (1.8)	

DM, diabetes mellitus; IFG, impaired fasting glucose.

**Table 1-2:** Participant characteristics and glycemic status.

		Normo-glycaemia	IFG+ DM	*p* (Mann-Whitney U test)
Anthropometrics	BMI (kg/cm^2^)	23.62(21.45, 25.95)	24.77(22.49, 27.07)	<0.001
	WC (cm)	82.27(73.33, 90.00)	86.67(80.00, 93.00)	<0.001
Lipid	TC (mmol/L)	4.84 (4.29, 5.46)	5.11(4.49, 5.11)	<0.001
	TG (mmol/L)	1.30(0.85, 2.06)	1.96(1.21, 3.11)	<0.001
	HDL (mmol/L)	1.10(1.02, 1.20)	1.10(0.96, 1.21)	0.06
	LDL (mmol/L)	3.39(2.96, 4.19)	3.41 (2.93, 4.16)	0.09
Lipid ratios	TC/HDL	4.54(3.85, 5.04)	4.76(4.17, 5.35)	<0.001
	LDL/HDL	3.15(2.62, 3.87)	3.18(2.60, 3.89)	0.95
	TG/HDL	2.67(1.80, 4.33)	4.05(2.49, 6.81)	<0.001

Data shown in median (interquartile range).

BMI, body mass index; DM, diabetes mellitus; HDL, high-density lipoprotein; LDL, low-density lipoprotein; TC, total cholesterol; TG, triglyceride; WC, waist circumference.

As shown in **Table 2**, family history of diabetes was significantly associated with hyperglycemia in the multivariable logistic regression analysis (odds ratio [OR] for family history ‘++’ with reference to ‘-’, 4.38, 95% confidence interval [CI]: 2.80–6.88; OR for family history ‘+’ with reference to ‘-’, 1.40, 95% CI: 1.16–1.69). The following variables were also significantly associated with hyperglycemia: male, age, higher BMI, higher income, preference for sweetened food, abnormal ECG, and fatty liver. In addition, for 1 unit (mmol/L) increase in TC and TG, the odds ratio for the hyperglycemia was 1.26 (95% CI: 1.17–1.37) and 1.10 (95% CI: 1.06–1.14), respectively. Regular physical exercise was found as a protective factor of hyperglycemia (OR=0.74, 95% CI: 0.63–0.88).

**Table 2:** Result of logistic regression analysis for IFG and DM (n=1125).

Parameters		*P*	OR	95% CI		Parameters		*P*	*OR*	95% CI	
				Lower	Upper					Lower	Upper
Family history of diabetes	>1 family member (++)	<0.001	4.38	2.8	6.88	Educational level	≥College degree	0.14	0.83	0.66	1.06
	1 family member (+)	<0.001	1.4	1.16	1.69		High school or equivalent	0.08	0.84	0.68	1.02
	None (-)	Reference group					< High school	Reference group			
Sex	Male	<0.001	1.93	1.60	2.33	ECG	Abnormal	0.02	1.26	1.04	1.52
	Female	Reference group					Normal	Reference group			
Fatty liver	Yes	0.004	1.25	1.08	1.46	Preference for sweetened food	Yes	<0.001	1.35	1.17	1.56
	No	Reference group					No	Reference group			
Age	≥ 60	<0.001	2.76	1.80	4.23	BMI	≥25	0.03	1.23	1.03	1.45
	50∼59	<0.001	2.17	1.80	2.62		23.0 ∼ 24.9	0.28	1.11	0.92	1.35
	40∼49	0.6	0.95	0.81	1.13		18.5 ∼ 22.9	Reference group			
	<40	Reference group					<18.5	0.13	0.67	0.41	1.12
Income level	≥¥3,000	0.04	1.31	1.01	1.70	Physical exercise	Regular	0.001	0.74	0.63	0.88
	¥2,000 -¥3,000	0.28	1.12	0.91	1.38		Occasional	0.28	0.89	0.73	1.08
	<¥2,000	Reference group					Never/Less	Reference group			
Smoking status	Smoker	0.5	1.07	0.89	1.28	Drinking status	Hazardous	0.86	1.02	0.84	1.24
	Passive smoker	0.65	0.95	0.78	1.17		Moderate	0.69	0.94	0.71	1.25
	Ex-smoker	0.34	1.15	0.86	1.54		Standard	0.94	0.99	0.79	1.23
	Non-smoker	Reference group					Non-drinking	Reference group			
Lipid	TC	<0.001	1.26	1.17	1.37	Others	LDL/HDL	0.5	0.99	0.95	1.02
	TG	<0.001	1.10	1.06	1.14		TG/HDL	0.27	0.95	0.88	1.04
	HDL	0.52	1.00	0.98	1.01		TC/HDL	0.39	0.97	0.91	1.04
	LDL	0.39	1.00	0.96	1.01		WC	0.61	0.99	0.99	1.00

BMI, body mass index; CI, confidence interval; DM, diabetes mellitus; ECG, electrocardiogram; IFG, impaired fasting glucose; HDL, high-density lipoprotein; LDL, low-density lipoprotein; OR, odds ratio; TC, total cholesterol; TG, triglyceride; WC, waist circumference.

The best cutoff value of incidence of diabetes in the first-degree relatives was 9.55%, with the area under the ROC curve of 0.60 (95% CI; 0.57–0.63, *P* < 0.001), 34.0% sensitivity and 85.2% specificity.

### 3.3 Factors associated with diagnosed T2DM

After conducting the bivariate analyses and multivariable analysis of 477 patients with T2DM (282 previously diagnosed patients and 195 newly identified patients), the following variables showed significant associations with previously diagnosed T2DM (*P*<0.001, **Table 3**): age (OR, 2.00; 95% CI, 1.56–2.57), positive family history (OR, 2.42; 95% CI, 1.67–3.52), and higher BMI (OR, 1.29, 95% CI, 1.03–1.62). Other variables (income, preference for sweetened food, physical exercise) were not associated with the diagnosis status.

**Table 3:** Result of logistic regression analysis for T2DM (n=477).

Parameter	B	S.E.	Wald	*P*	Rude OR	Adjusted OR	95% confidence interval	
							Lower	Upper
Family history	0.89	0.19	21.514	<0.001	2.47	2.42	1.67	3.52
Age	0.69	0.13	29.408	<0.001	1.97	2.00	1.56	2.57
Income level	-0.02	0.18	.011	0.92	1.00	0.98	0.69	1.39
Preference for sweetened food	-0.41	0.25	2.814	0.09	0.65	0.66	0.41	1.07
Physical exercise	0.26	0.14	3.395	0.07	1.29	1.30	0.98	1.72
Body mass index	0.25	0.12	4.859	0.03	1.35	1.29	1.03	1.62
Constant	-3.08							

The predictive equation was calculated with the following logistic regression parameters: P = \frac{1}{1 + e^{-z}}, where z = -3.08 + [0.89 (Family history-group) + 0.69 (age-group)+ 0.25 (BMI-group)]. (1)Family history ‘++’=2; family history ‘+’=1; family history ‘-’=0. (2) ‘age≥ 60 years old’=3; ‘50-59 years old’=2; ‘40-49 years old’=1; ‘<40 years old’=0.(3) ‘BMI≥ 25kg/m^2^’=2; ‘23.0-24.9kg/m^2^’=1; ‘< 22.9kg/m^2^’=0.

**Figure 2:**
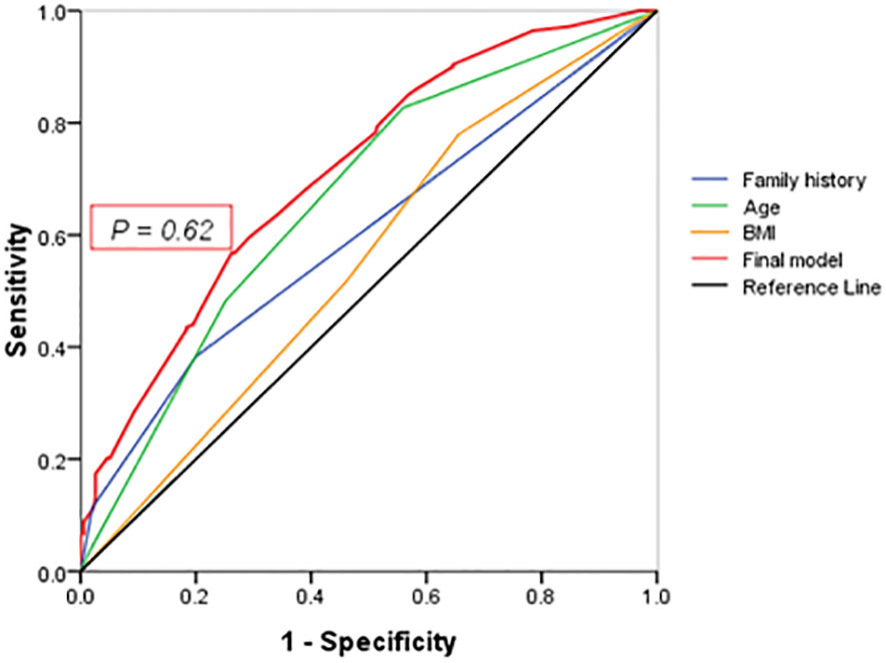
ROC curve. Points on the ROC curve means positive if it is greater than or equal to the probability value of 0.62 provided a sensitivity of 64% and a specificity of 29%. The area under the final model including all independent factors is 0.71(95% CI: 0.67–0.76). Areas for other independent factors are as follows: (1) AUC_Family history_ = 0.60(95% CI:0.61–0.71); (2) AUC_Age_ = 0.67(95% CI:0.61–0.71); (3) AUC_BMI_ = 0.60(95% CI:0.55–0.65).

The predictive equation was calculated with the following logistic regression parameters with 0.71 (95% CI: 0.67–0.76) of the area under the ROC curve (**Figure 2**), 64.0% of sensitivity and 29% of specificity (*P* < 0.001): *P* = \frac{1}{1 + e^{-z}}, where z = -3.08 + [0.89 (Family history-group) + 0.69 (Age-group)+ 0.25 (BMI-group)] (**Table 3**). Positive family history was associated with the diagnosis of T2DM, but not glucose level in the diagnosed patients. The best cutoff value of incidence of diabetes in the first-degree relatives was 9.55% (*P* < 0.001).

Among the 282 patients with previously diagnosed T2DM, no significant differences were found in glucose levels across different degree of family history (as depicted in **Table 4**).

**Table 4:** Family history and glucose level in diagnosed T2DM patients (n = 282).

Number of first-degree relatives with diabetes	Glucose ≥7.0 mmol/L, n (%)	Glucose <7.0 mmol/L, n (%)	*P*
>1	14 (43.8)	18 (56.2)	0.57
1	21 (27.6)	55 (72.4)	
0	70 (40.2)	104 (59.8)	

## 4 Discussion

Among people with T2DM, 40.9% T2DM had not been diagnosed and more than 1/3 of diagnosed ones weren’t well controlled. Research has found that modifiable lifestyles, such as routine dietary habits and physical activity, are also associated with glucose metabolism (20). Preference for sweetened food has been reported as a potential predictor for T2DM (21, 22). The intake of sugary drinks, desserts and other sweets has become a widely preferable lifestyle instead of alerting an excessive calorie intake (23). In this study, the preference for sweetened taste was identified as one of the independent risk factors for T2DM. Physical activity can help to slow down the progression of disease in individuals with prediabetes by increasing glucose uptake and utilization, improving insulin sensitivity, and protecting pancreatic β-cell function (24–27). Physical exercise of at least 30 minutes no less than 3 times per week showed a protective effect against glucose metabolic disorders. Other unmodifiable risk factors reported in the literature include age and sex, showing prevalence in men was 1.9 times as high as in women, and increased with age (over 50) (28–31).

**Figure 3:**
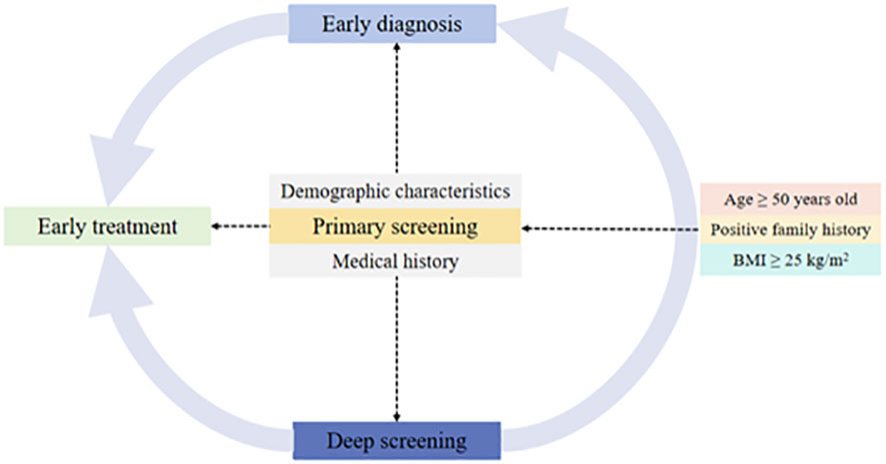
Targeting the people at high risk for diabetes and primary screening.

As for targeting the people at high risk for diabetes, we emphasize the significance of recording the family history of T2DM in first-degree relatives as well as age and BMI (**Figure 3**). In this study, the target population with positive family history (16.3%) accounted for 25% of diabetes. Specifically, people with one first-degree relative with T2DM had OR of 1.40 for T2DM, and the OR increased to 4.38 when more than one family member had T2DM. Other studies also confirmed the role of family history of diabetes depending on the number of affected relatives (32–34).

Moreover, we found that positive family history of diabetes was associated with diabetes being diagnosed but not with the glycemic control. Family history of diabetes is not only a risk factor for the disease, but also an indicator for detecting undiagnosed diabetes (35, 36). Certainly, the results are not always consistent. A previous study suggested higher familial risk of diabetes resulting in a worse glycemic control (37). Another study reported that positive family history of diabetes was significantly associated with good control (38). Our study suggested that family history of diabetes could provide a useful screening tool for detection and prevention of diabetes.

To develop a useful screening tool for detection and prevention of diabetes, numerous studies have preliminarily validated the generalizability of diabetic prediction models. For instance, a cohort study published in BMJ journal developed and validated three updated QDiabetes risk models with new risk factors including lipid-lowering drugs or mood-related medications and symptoms to quantify the absolute risk of T2DM (39). Moreover, it is difficult to make a perfect auto-tuning or morphing tool for accurate prediction of multifactorial diseases. An external validation of the 10-year risk prediction model revealed that the AUC levels from TLGS data with a younger population (0.79) were slightly higher than those from the development data (0.789) (40). Yochai Edlitz et al. (41) have suggested that further refinement of the feature coefficients may be necessary for their prediction models to be applicable to diverse populations with a prevalence of T2DM higher than 1.79%, despite the ideal diagnostic significance of the obtained model (AUC: 0.81, 95% CI: 0.77–0.84). In our study, the intersection of curves also indicates that the value of each independent factor has little significance, and the integrated model can better coordinate the diagnostic value of each independent factor.

### 4.1 Significance and limitations

Though primary care is essential for early prophylaxis, early diagnosis and early treatment for a multitude of diabetes patients, the implementation strategy remains discussed. Besides, WHO and the ADA recommend screening only among high-risk, asymptomatic individuals. Consistently, a family history of diabetes is one of the variables included in screening tools as an independent contributor to risk (42, 43). As for targeting the people at high risk for diabetes, we emphasize the importance of recording the family history of T2DM in first-degree relatives as well as age, sex, and BMI. In this study, the target population with positive family history (16.3%) accounted for 25% of diabetes. For the prevention and control of diabetes, positive family history means not only the genetic factors of T2DM, but also similar unhealthy lifestyles. It is worth mentioning that we did not consider second-degree relatives in this survey. This was because more than half did not respond to this question in the pre-survey of 100 participants and also because of the possibility for the recalling bias. On the contrary, family history in first-degree relatives is one piece of easily accessible genetic information. Additionally, it is imperative to bolster high-quality research in the realm of diabetes, focusing on innovative drug development, health economics, and the enhancement of healthcare quality (44, 45).

This study took into account missing samples and expanded the sample size by 20%. However, it is not ruled out that the missing information is caused by diabetic patients’ fear of revealing their condition and refusing to check relevant items. Such missing information may occur non-randomly and cover up the association between family history and diabetes, which may lead to bias in the study results. It is more worthwhile to analyze the reasons separately in order to provide the direction for the health education of people who avoid health screening.

Because this was a cross-sectional observational study, causal inference cannot be concluded. In addition, although we analyzed numerous variables, there remains a possibility of unmeasured confounders. Besides, diabetes duration and complications should be studied further to explore the specific influence of family-based health education.

### 4.2 Clinical implications

Despite of the lengthy battle against diabetes, it’s still worth thinking about many root problems. For example, most people are afraid of incurable disease or emergency, but tend to ignoring unhealthy lifestyles or positive family history related to chronic non-communicable diseases under the COVID-19 pandemic. The primary medical screening policy would play a prominent role in solving this “frog in the boiling water” problem.

Our findings have emphasized the importance of recording the family history of T2DM in first-degree relatives for diagnosis of diabetes, especially when the proportion of positive in the family exceeds 9.55%. As for dynamic screening, original records of community health survey could help to lock and classify the target population according to the positive number of first-degree relatives. As a result, the primary medical resources would be more effectively and efficiently used, followed by early diagnosis and early treatment in clinic.

## 5 Conclusions

Family history of diabetes was independently associated with glucose dysfunction. Classification by the first-degree relatives with diabetes is important for targeting high-risk population. Simultaneously, positive family history of diabetes was associated with diabetes being diagnosed but not with the glycemic control in patients who had been diagnosed. Enhancing the early diagnosis linkage with positive family history of diabetes is considerably imperative for high proportions of undiagnosed T2DM.

## Data availability statement

The original contributions presented in the study are included in the article/supplementary material. Further inquiries can be directed to the corresponding author.

## Ethics statement

The studies involving humans were approved by China-Japan Union Hospital of Jilin University Ethics Committee for Human Subjects. The studies were conducted in accordance with the local legislation and institutional requirements. The participants provided their written informed consent to participate in this study. Written informed consent was obtained from the individual(s) for the publication of any potentially identifiable images or data included in this article.

## Author contributions

ZH: Writing – original draft, Writing – review & editing. HaY: Writing – review & editing. HiY: Writing – review & editing. HL: Writing – review & editing. XW: Writing – original draft, Writing – review & editing.
